# The effects of exercise treatment on learning and memory ability, and cognitive performance in diet-induced prediabetes animals

**DOI:** 10.1038/s41598-020-72098-0

**Published:** 2020-09-14

**Authors:** Mluleki Luvuno, Andile Khathi, Musa V. Mabandla

**Affiliations:** School of Laboratory Medicine and Medical Sciences, College of Health Sciences, University of KwaZulu-Natal, Private Bag X54001, Durban, South Africa

**Keywords:** Neuroscience, Physiology, Endocrinology

## Abstract

Changes associated with cognitive function in the high-fat high-carbohydrate diet-induced prediabetes animal model and effect of exercise remain unclear. Rats were randomly assigned to the following groups (n = 6): non-diabetic (ND), prediabetic (PD), intermittent exercising PD (PD + IE) and regular exercising PD (PD + RE). After exercise cessation, oral glucose tolerance (OGT), Novel Object Recognition Test (NORT) and Morris-Water Maze (MWM) tests were performed to assess cognitive function. After sacrifice, malonaldehyde, glutathione peroxidase, interleukin-1β and dopamine concentration in the prefrontal cortex (PFC) and hippocampus were measured. Impaired OGT response in PD animals was accompanied by poor performance on behavioural tasks. This was associated with increased oxidative stress markers and impaired dopamine neurotransmission as evidence by elevated dopamine concentration in the PFC and hippocampal tissue. Improved OGT response by exercise was coupled with improved performance on behavioural tasks, oxidative stress markers and increased interleukin-1β concentration. In regular exercise, this was further coupled with improved dopamine neurotransmission. Cognitive function was affected during prediabetes in animals. This was partly due to oxidative stress and impaired dopamine neurotransmission. Both intermittent and regular exercise improved cognitive function. This was partly mediated by improved glucose tolerance and oxidative stress as well as a subclinical increase in interleukin-1β concentration. In regular exercise, this was further mediated by improved dopamine neurotransmission.

## Introduction

Cognitive dysfunction has huge economic and social costs not only for those affected but for their caregivers as well^1,2^. Once started, cognitive dysfunction advances with age and most of the time when this has been identified the changes would have reached an irreversible state. Diabetes and its related derangements have been strongly linked to cognitive decline in the ageing population^3^. Both diabetes and cognitive decline increased alarmingly following a rise in unhealthy feeding patterns and sedentary lifestyle^4,5^. Therefore, a need to deeply understand the relationship between diabetes and cognitive decline, early identification of cognitive problems and effective implementation of therapeutic methods is increasingly important^6^.

Dyslipidaemia, hypertension, hyperglycaemia, hypoglycaemia and inflammation common to diabetes have a great association with cognitive impairments^7^. However, most cases of cognitive impairment in the ageing population have been associated with constant high blood glucose and impaired glucose tolerance^8,9^. An increased concentration of proinflammatory cytokines in the circulation has been shown to lead to dysregulation of the blood–brain-barrier^10^. Barrier dysfunction increases the permeability of brain microvascular endothelial cells and exposes the brain to increased blood glucose concentration^11^. Higher blood glucose concentration in the brain elicit adverse effects such as inflammation and oxidative stress which compromises cognitive function^12^. This manifests in poor cognitive performance and interferes with many cognitive tasks of daily living^13^.

High-fat and or high carbohydrate diet have been implicated in the alteration of the brain regions essential for learning, memory and cognition function^14^. In our laboratory, prolonged ingestion of a high-fat high carbohydrate (HFHC) diet led to the development of prediabetes and its related derangements in relatively sedentary animals^15^. These derangements include hyperglycaemia, dyslipidaemia, insulin resistance, oxidative stress, inflammation, and hypertension. All of these have been mechanistically linked to cognitive impairments^7^. However, whether these metabolic derangements in the HFHC-induced prediabetes are accompanied by changes in learning and memory activity and general cognitive function in the brain is yet to be elucidated. Identifying if the cognitive problems start to happen as early as at a prediabetes state following ingestion of an HFHC diet can open avenues to effectively curb cognitive problems as early as possible.

Physical exercise has been shown to alleviate diabetes-related derangements and convey protective effects against neurodegenerative diseases^16–20^. However, whether physical exercise can ameliorate cognitive deficits that may have occurred following prolonged ingestion of an HFHC diet in prediabetic animals remain unknown. Therefore, the present study looks at selected metabolic, behavioural and neurotransmitter changes related to cognitive function in HFHC diet-induced prediabetes in sedentary animals and the ameliorative effects of physical exercise. One of the main advantages of exercise is that it can be customised according to the patient’s needs. As such a patient may only afford intermittent training while required to do regular training while the other may afford regular exercise training. This depends on a patient’s daily schedule and the severity of the condition. Hence, intermittent and regular exercise will be used as a form of treatment in the present study.

## Results

### OGT response

**Figure 1:**
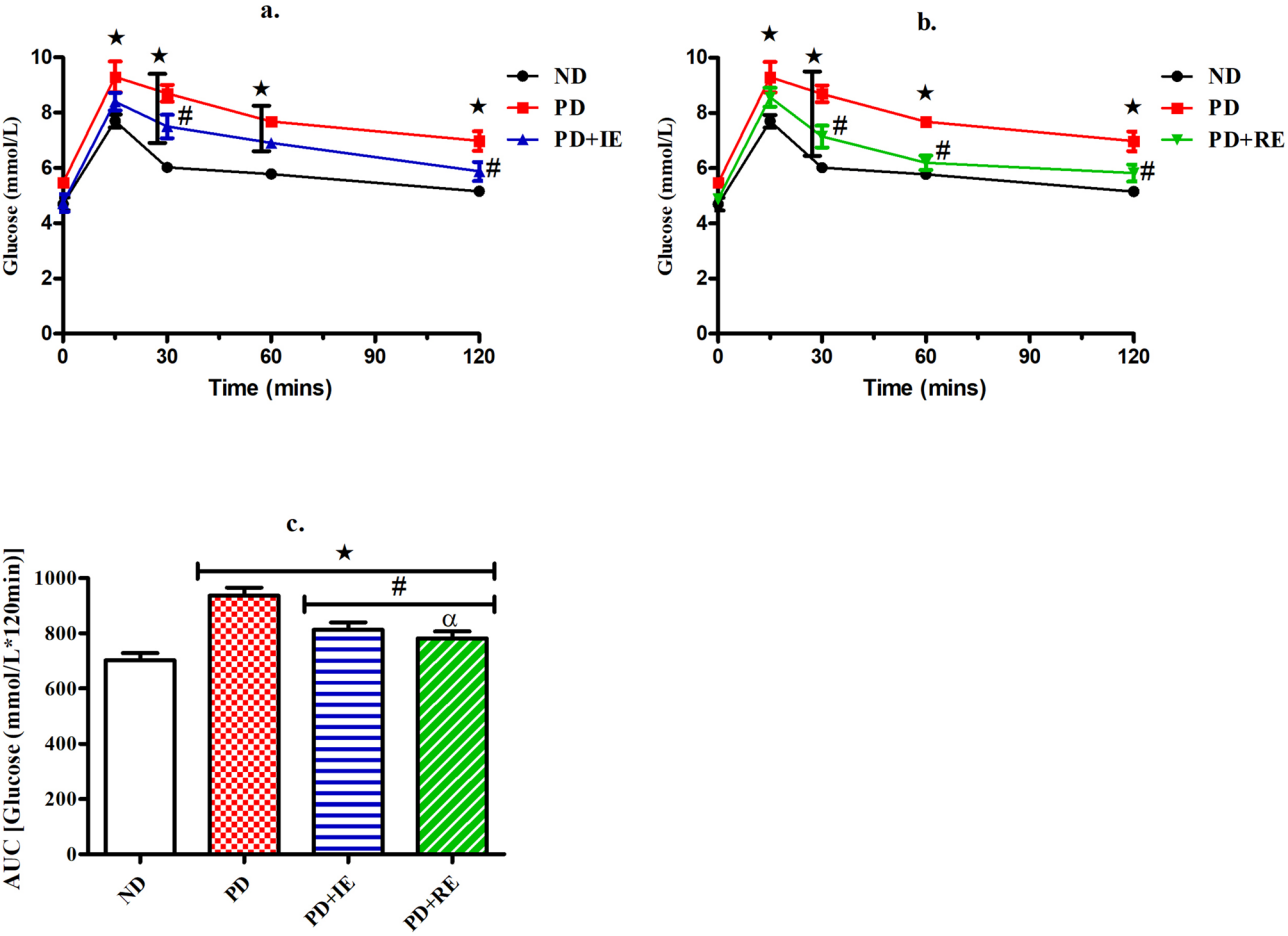
OGT response (**a**, **b**) and AUC_glucose_ values (**c**) to glucose loading of ND, PD, PD + IE and PD + RE animal groups (n = 6, per group) two weeks after exercise cessation. Values are presented as means ± SEM. *p < 0.05 denotes comparison with ND group; ^**#**^*p* < 0.05 denotes comparison with PD group, ^α^*p* < 0.05 denotes comparison with the PD + IE group.

The OGT response of ND, PD, PD + IE and PD + RE animal groups (n = 6, per group) was assessed two weeks post-exercise cessation. There was a PD effect on blood glucose concentration starting from 15 min post bolus injection to the end of the test *(ND vs. PD, *p* < 0.05, Fig. 1a,b). This effect was also present in the exercised groups at 30 min post bolus injection *(ND vs. PD + IE, *p* < 0.05, Fig. 1a) and (ND vs. PD + RE, *p* < 0.05, Fig. 1b). Similarly, this effect was present in the PD + IE group at 60 min post bolus injection *(ND vs. PD + IE, *p* < 0.05, Fig. 1a). There was an exercise effect on blood glucose concentration at the 30 min observation point ^#^(PD vs. PD + IE, *p* < 0.05, Fig. 1a) and ^#^(PD vs. PD + RE, *p* < 0.05, Fig. 1b). This effect was present on all subsequent time points in the PD + RE group ^#^(PD vs. PD + RE, *p* < 0.05, Fig. 1b) but was only present at the 120 min observation point in the PD + IE group ^#^(PD vs. PD + IE, *p* < 0.05, Fig. 1a). The corresponding AUC_glucose_ values further show the PD effect *(ND vs. PD, ND vs. PD + IE, ND vs. PD + RE, *p* < 0.05, Fig. 1c), while the exercise effect was present in the PD groups ^#^(PD vs. PD + IE, PD vs. PD + RE, *p* < 0.05, Fig. 1c). A regular exercise effect was present in the exercised groups ^α^(PD + IE vs. PD + RE, *p* < 0.05, Fig. 1c).

### NORT task

**Figure 2:**
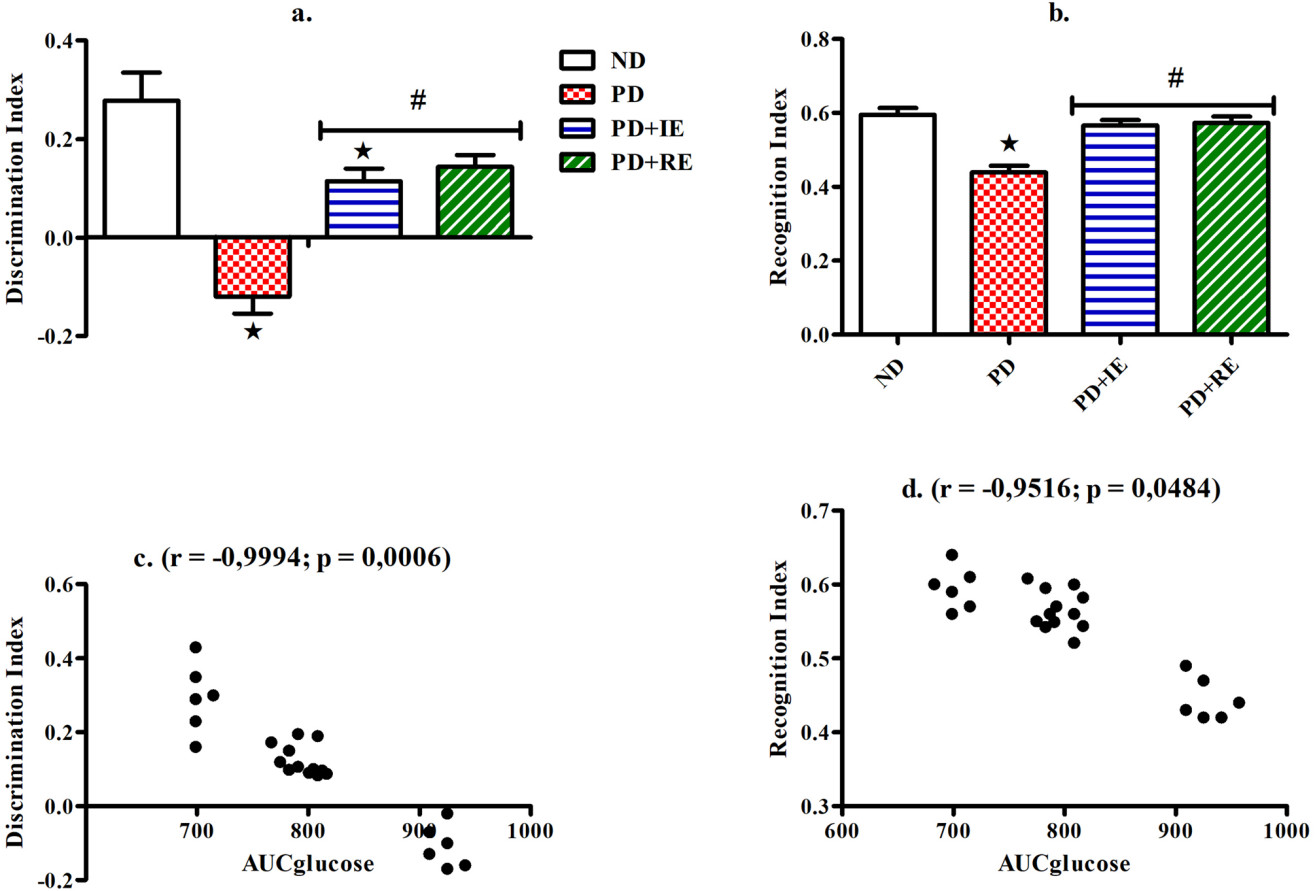
Discrimination Index (**a**) and Recognition Index (**b**) during the NORT task of the ND, PD, PD + IE and PD + RE animal groups (n = 6, per group) one week following exercise cessation. Correlation of the AUC_glucose_ with Discrimination Index (**c**) and Recognition Index (**d**) from the NORT task with individual data across the groups (groups = 4 × number of animals (6, per group) = 24). Values are presented as means ± SEM. *p < 0.05 denotes comparison with ND group; ^**#**^*p* < 0.05 denotes comparison with PD group.

Discrimination Index (a.) and Recognition Index (b.) of the NORT task in the ND, PD, PD + IE and PD + RE groups (n = 6, per group) one-week post-exercise cessation was calculated. Correlation analysis of the AUC_glucose_ with discrimination index (c.) and recognition index (d.) was also performed with individual data across the groups (groups = 4 × number of animals (6, per group) = 24). There was a PD effect on discrimination index value *(ND vs. PD, ND vs. PD + IE, *p* < 0.05, Fig. 2a), while exercise effect was present in the PD groups ^#^(PD vs. PD + IE, PD vs. PD + RE, *p* < 0.05, Fig. 2a). There was also a PD effect on recognition index value *(ND vs. PD, *p* < 0.05, Fig. 2b). Exercise effect was present in the PD groups ^#^(PD vs. PD + IE, PD vs. PD + RE, *p* < 0.05, Fig. 2b). The AUC_glucose_ negatively correlated with the discrimination index of the NORT (r = − 0.9994, *p* = 0.0006, Fig. 2c.). The same effect was present with the recognition index of the NORT (r = − 0.9516, *p* = 0.0484 Fig. 2d.).

### MWM task

**Figure 3:**
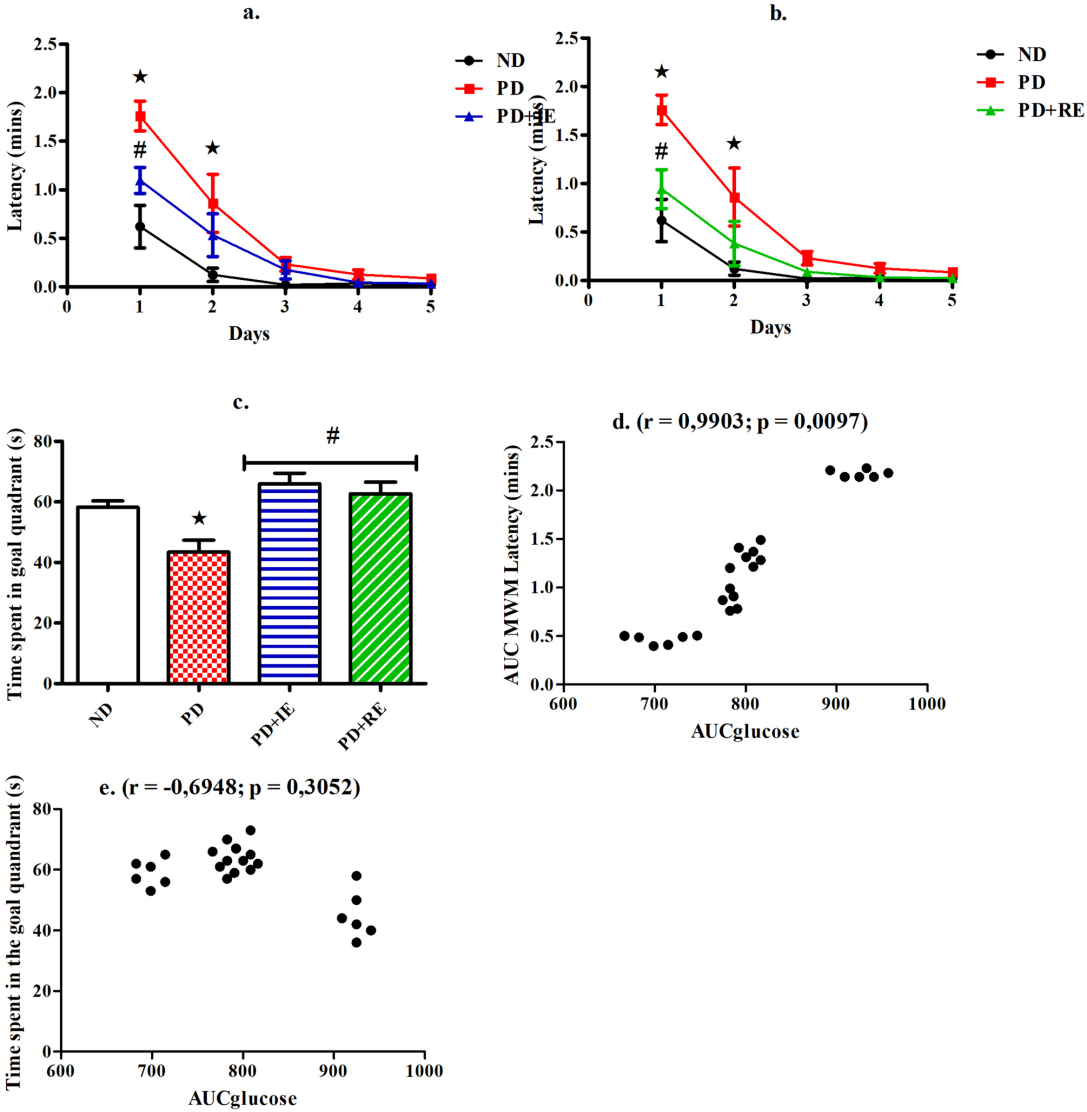
Latency (**a**, **b**) during MWM task and time spent in the goal quadrant (**c**) during the MWM probe test in the ND, PD, PD + IE and PD + RE groups (n = 6, per group) one week following exercise cessation. Correlation of the AUC_glucose_ with the AUC_MWM latency_ (**d**) and time spent in the goal quadrant (**e**) from the MWM task with individual data across the groups (groups = 4 × number of animals (6, per group) = 24). Values are presented as means ± SEM. *p < 0.05 denotes comparison with ND group; ^**#**^*p* < 0.05 denotes comparison with PD group.

The graphs below denote the measurement of latency (a. and b.) MWM test and time spent in the goal quadrant (c.) during the MWM probe test in the ND, PD, PD + IE and PD + RE groups (n = 6, per group) one week following exercise cessation. Correlation analysis of the AUC_glucose_ glucose with the AUC_MWM latency_ (d.) and time spent in the goal quadrant (e.) during the MWM task was also performed with individual data across the groups (groups = 4 × number of animals (6, per group) = 24). A PD effect was present on days 1 and 2 on latency *(ND vs. PD, *p* < 0.05, Fig. 3a/b). An exercise effect on latency was present on day 1 in the PD groups ^#^(PD vs. PD + IE, *p* < 0.05, Fig. 3a) and ^#^(PD vs. PD + RE, *p* < 0.05, Fig. 3b). There was a PD effect on time spent in the goal quadrant *(ND vs. PD, *p* < 0.05, Fig. 3c) while an exercise effect was present in the PD groups ^#^(PD vs. PD + IE, PD vs. PD + RE, *p* < 0.05, Fig. 3c). The AUC_glucose_ positively correlated with AUC latency of the MWM task (r = 0,9,903, *p* = 0.0097, Fig. 3d.). However, there was no effect between the 2 h OGT and the time spent in the goal quadrant during the MWM probe test (r = − 0.6948, *p* = 0.3052, Fig. 3e.).

### Oxidative stress markers

**Figure 4:**
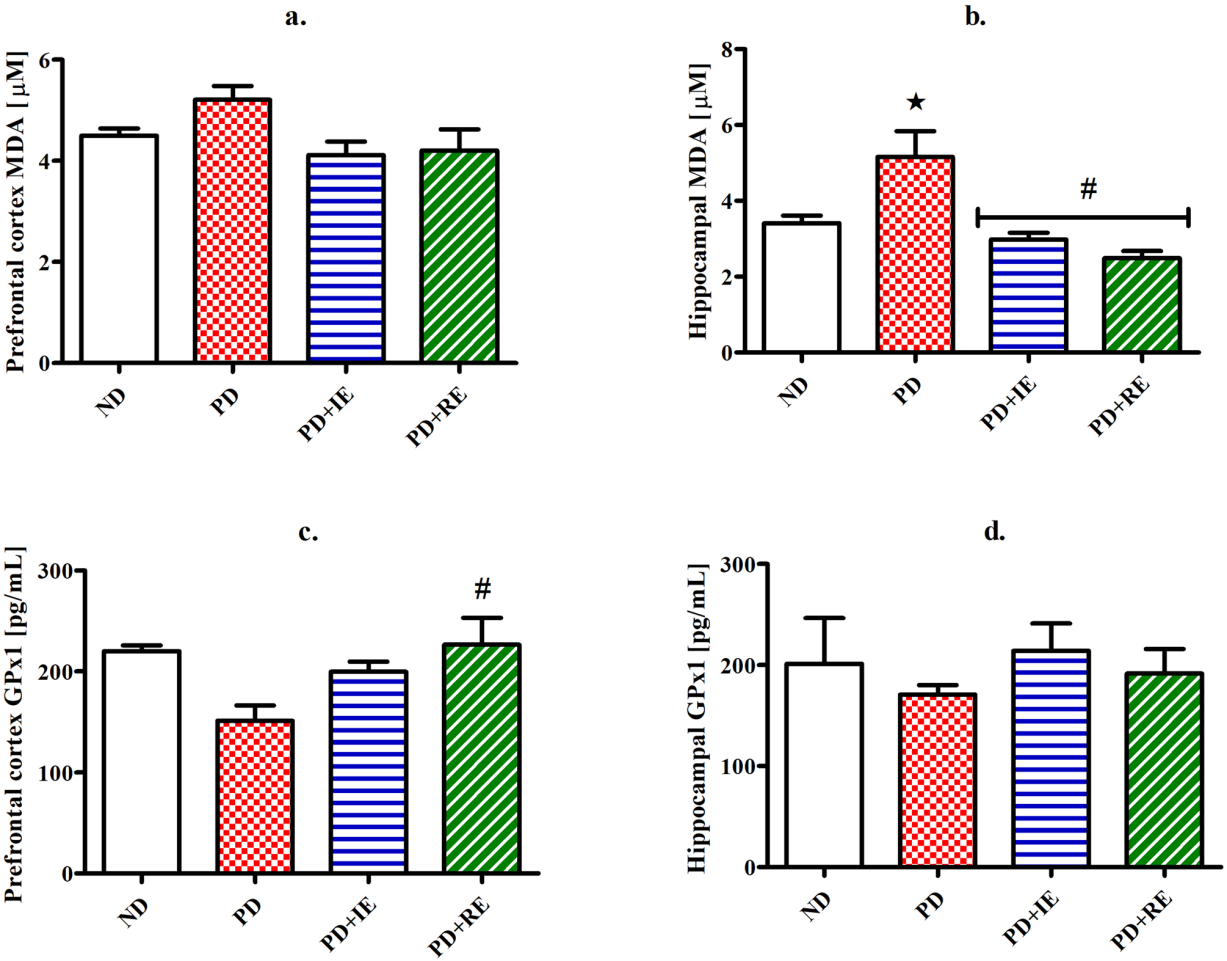
MDA concentration in the PFC (**a**) and hippocampus (**b**) as well as GPx1 concentration in the PFC (**c**) and hippocampus (**d**) of the ND, PD, PD + IE and PD + RE groups (n = 6, per group) two weeks following exercise cessation. Values are presented as means ± SEM. *p < 0.05 denotes comparison with ND group; ^#^*p* < 0.05 denotes comparison with PD group.

MDA concentration in the PFC (a.) and hippocampal (b.) brain tissues, as well as GPx1 concentration in the PFC (c.) and hippocampal (d.) brain tissues of the ND, PD, PD + IE and PD + RE groups (n = 6, per group) two weeks following exercise cessation, were measured. There was no PD effect on MDA concentration in the PFC tissue (ND vs. PD, Fig. 4a). There was also no exercise in the present in the PD groups (PD vs. PD + IE, PD vs. PD + RE, Fig. 4b). There was a PD effect on MDA concentration in hippocampal tissue *(ND vs. PD, *p* < 0.05, Fig. 4b). However, an exercise effect was present in the PD groups ^#^(PD vs. PD + IE, PD vs. PD + RE, *p* < 0.05, Fig. 4b). A regular exercise effect was present in the PFC on GPx1 concentration ^#^(PD vs. PD + RE, *p* < 0.05, Fig. 4c). There was no PD effect on GPx1 concentration in the hippocampal tissue (ND vs. PD, Fig. 4d). There was also no exercise in the present in the PD groups (PD vs. PD + IE, PD vs. PD + RE, Fig. 4d).

### IL-1β concentration

**Figure 5:**
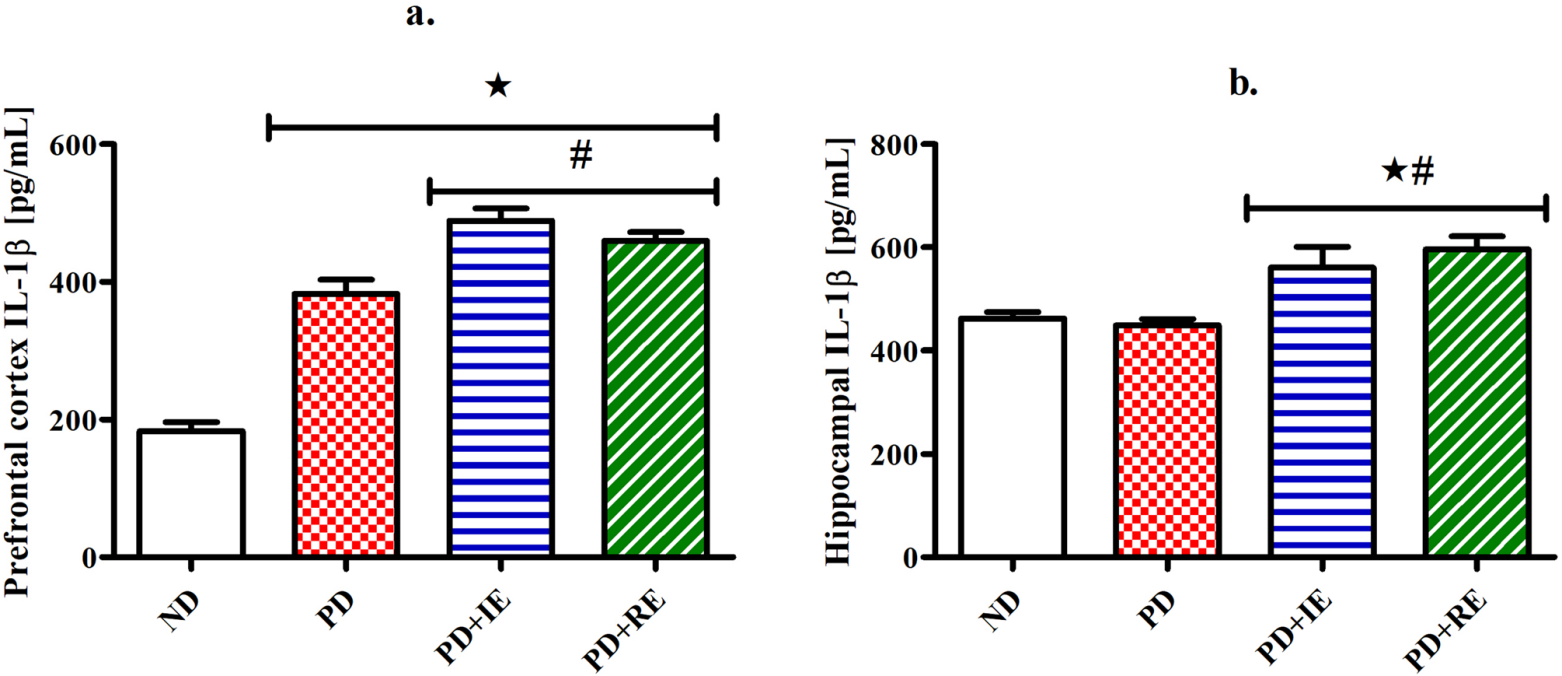
PFC (**a**) and hippocampal (**b**) IL-1β concentration in the ND, PD, PD + IE and PD + RE groups (n = 6, per group) two weeks following exercise cessation. Values are presented as means ± SEM. *p < 0.05 denotes comparison with ND group; ^**#**^*p* < 0.05 denotes comparison with PD group.

IL-1β concentration in the PFC (a.) and hippocampus (b.) of the ND, PD, PD + IE and PD + RE groups (n = 6, per group) two weeks following exercise cessation was measured. There was a PD effect on PFC IL-1β concentration *(ND vs. PD, ND vs. PD + IE and ND vs. PD + RE, *p* < 0.05, Fig. 5a). An exercise effect was present in the PD groups ^#^(PD vs. PD + IE AND PD vs. PD + RE, *p* < 0.05, Fig. 5a). Combined PD and exercise resulted in elevated hippocampal IL-1β concentration *(ND vs. PD + IE, ND vs. PD + RE, *p* < 0.05, Fig. 5b) as well as ^#^(PD vs. PD + IE, PD vs. PD + RE, *p* < 0.05, Fig. 5b).

### Dopamine (DA) concentration

**Figure 6:**
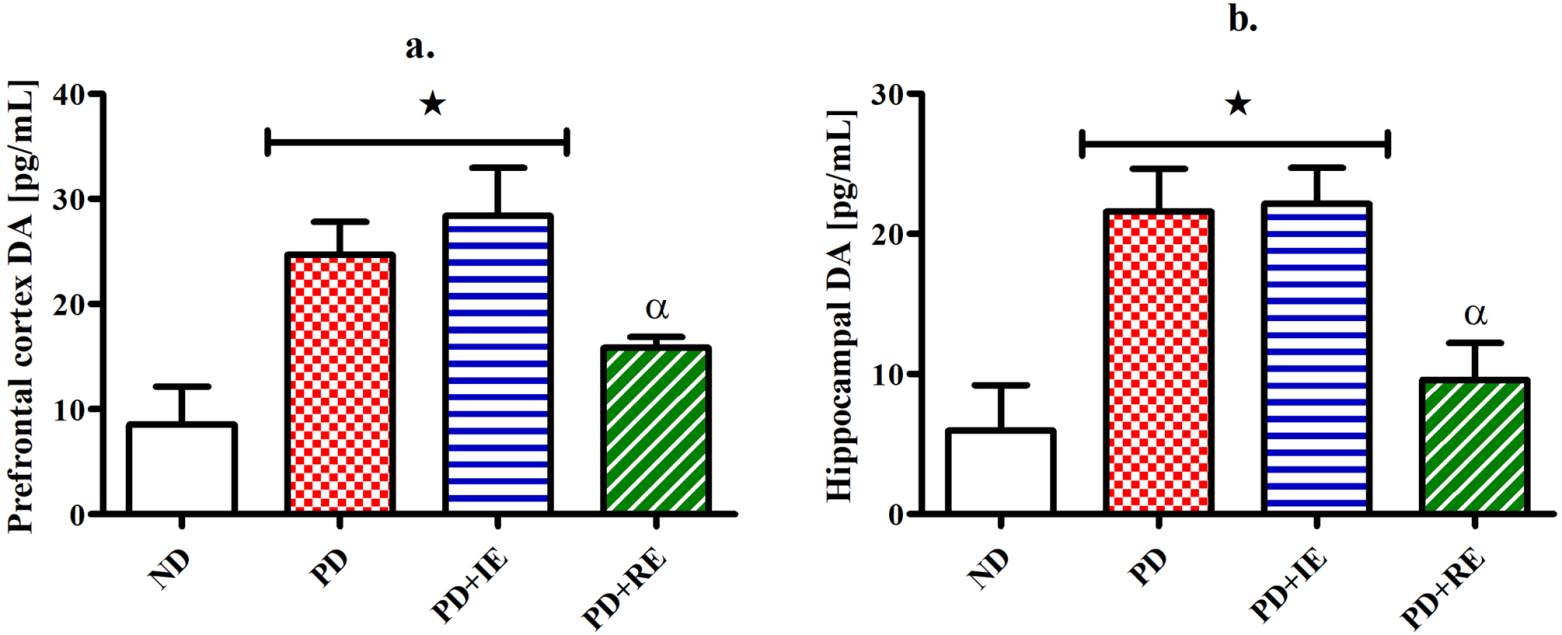
PFC (**a**) and hippocampal (**b**) DA concentration in the ND, PD, PD + IE and PD + RE groups (n = 6, per group) two weeks following exercise cessation. Values are presented as means ± SEM. *p < 0.05 denotes comparison with ND group, ^**#**^*p* < 0.05 denotes comparison with PD group, ^α^*p* < 0.05 denotes comparison with the PD + IE group.

DA concentration in the PFC (a) and hippocampal (b) brain tissue of the ND, PD, PD + IE and PD + RE groups (n = 6, per group) was measured two weeks following exercise cessation. PD and PD + IE groups had elevated DA concentration in the PFC when compared to the ND group *(ND vs. PD, ND vs. PD + IE, *p* < 0.05, Fig. 6a). There was a regular exercise effect in the exercised groups ^α^(PD + IE vs. PD + RE, *p* < 0.05, Fig. 6a). Similarly, PD and PD + IE groups had elevated DA concentration in hippocampal tissue *(ND vs. PD, ND vs. PD + IE, *p* < 0.05, Fig. 6b). And a regular exercise effect was present in the exercised groups ^α^(PD + IE vs. PD + RE, *p* < 0.05, Fig. 6b).

## Discussion

Findings of the present study demonstrate that the metabolic derangements following prolonged ingestion of an HFHC diet in prediabetic and sedentary animals were accompanied by metabolic, behavioural and brain changes associated with impaired cognitive function. Both intermittent and regular exercise ameliorated the changes that were associated with cognitive impairment in these animals. This reveals that the derangements that underpin cognitive impairment in the ageing population start to occur as early as at a prediabetes stage. The study shows that timely intervention before the cognitive impairment is irreversible can curb the increasing rise in cognitive problems amongst the ageing population.

The present study shows that changes in glucose tolerance correlated with changes in NORT and MWM task thus suggesting a link between glucose tolerance and cognitive function. An impaired glucose tolerance response following a 2-h OGT test found in the PD group was accompanied by reduced insulin sensitivity in the previous study^15^. High-fructose or high-fat feeding has been shown to impair neural insulin signalling in the core brain areas supporting learning and memory and general cognitive function^21–23^. Therefore, we speculate that chronic ingestion of the HFHC diet may have also led to alterations in insulin signalling in these brain areas. This may then lead to reduced processing speed and executive function; impaired learning and memory and cognitive decline^24,25^. The NORT and MWM task findings of the present study are in support of this speculation. The negative discrimination and lower recognition index values during the NORT task in the PD group suggests an inability to distinguish between a familiar and a novel object. The increased latency time to find a hidden platform and less time spent in the goal quadrant during the MWM task by the PD group suggests poor spatial learning and memory activity. These behavioural findings may be a manifestation of altered cognitive function.

Previous studies have mechanistically linked neurodegenerative conditions to diabetes-related derangements such as oxidative stress and inflammation^12,26^. Findings of the present study are in support of this. This is shown by the tendency of elevated MDA concentration and reduced GPx1 concentration in the PFC and hippocampal tissue of the PD group. This may increase susceptibility to lipid peroxidation in the tissues and contribute to cognitive impairment^5,27–34^. Chronic unabated elevation in IL-1β concentration and reduction in DA concentration have been shown to contribute to cognitive impairment^35–38^. However, the present study shows controversial findings though this did not affect the behavioural performance of the PD group. PFC IL-1β concentration was elevated while there was no change in the hippocampal IL-1β concentration. On the other hand, both PFC and hippocampal DA concentration were elevated. This is in contrast with studies that show that an increase in DA concentration during the MWM task enhance spatial learning^39^. Several studies have shown that higher DA levels during the oxidative stress-related brain disorders may be due to impaired dopamine reuptake^40–42^. Furthermore, it has been shown that high-fat diet-induced obesity results in reduced DA reuptake without altering DA transporter gene expression^43^. Therefore, higher DA concentration in the PD group could suggest problems with DA neurotransmission^44^.

One week after exercise cessation, there was an improved glucose tolerance in both the PD + IE and PD + RE groups despite continued exposure to an HFHC diet suggesting a lasting effect of exercise on glucose tolerance. This can be attributed to improved insulin sensitivity by exercise^17,45^. This was accompanied by improved performance in the NORT and MWM tasks in the exercised groups. This suggests that exposure to exercise had lasting effects on cognitive function. This is supported by the studies which show that exercise enhances object recognition memory and increases fine discrimination^46–48^. Better performance on the cognitive tasks by the exercised groups co-existed with a tendency of improved oxidative stress markers. Further to ameliorating metabolic derangements associated with cognitive impairment, exercise could have also achieved better cognitive performance by increasing dendritic complexity, resting-state perfusion and angiogenesis^18,49,50^.

Though the IL-1β concentration findings in the PD group were controversial, there was a general increase in the PFC and hippocampal tissue IL-1β concentration following exercise. Studies show that while a chronic unabated elevation in IL-1β concentration can be deleterious to the brain, an increase in IL-1β concentration is important for tissue repair, cellular defence and thus may protect against tissue injury in the brain^51^. Since the IL-1β concentration findings in the exercised animals were accompanied by improved performance on the cognitive tasks, we speculate that the increased IL-1β concentration following exercise conveyed neuroprotective effects. This is supported by a study that showed that IL-1β facilitates learning and spatial memory tasks in the MWM test^52^. Studies have also shown that exercise induces a subclinical inflammatory response that is mediated in part by increased concentration of proinflammatory cytokines including IL-1β^53,54^. A week after exercise termination, PD + IE and PD + RE groups showed different effects on DA concentration in the PFC and hippocampal tissue without affecting behavioural performance. The PD + RE group lowered the DA concentration while there was no change in DA concentration of the PD + IE group compared to the PD group. We have previously mentioned that the higher DA concentration in the PD group than the ND group could be due to impaired DA reuptake^40–43^. Therefore, we speculate that regular exercise improved DA reupdate than intermittent exercise. This can be attributed to the amount of time devoted to each type of exercise. A study shows that exercise improves motor and cognitive impairment in Parkinson’s disease partly by increasing DA neurotransmission in the corticostriatal circuits^19^. Therefore, findings of the exercised animals suggest that regular exercise improved DA neurotransmission to a greater degree than intermittent exercise. This can be attributed to a degree at which each exercise type increase the binding affinity between DA and its receptor^55^.

## Conclusion

Prolonged ingestion of the HFHC diet and sedentary lifestyle led to impaired OGT, poor performance on the NORT and MWM tasks which was accompanied by a tendency of increased oxidative stress markers and impaired DA neurotransmission in the PFC and hippocampal tissue. Exposure to exercise, whether intermittently or regularly, improved prediabetes induced learning, memory and general cognitive impairment. This was mediated in part by improved OGT and oxidative stress markers as well as a subclinical increase in IL-1β concentration from the PFC and hippocampus in the exercised animals. In regular exercise, this was further mediated by improved DA neurotransmission. Therefore, exercise ameliorated prediabetes induced effects on brain function and the lasting exercise effects do not dissipate immediately following exercise cessation.

## Materials and methods

### Chemicals

All chemicals and reagents were of analytical grade and were purchased from standard commercial suppliers.

### Animals

Male Sprague–Dawley rats (150–180 g) bred and housed in the Biomedical Research Unit of the University of KwaZulu-Natal were used in the study^15^. The animals were maintained under standard laboratory conditions of constant temperature (22 ± 2 °C), CO_2_ content (< 5,000 p.m.), relative humidity (55 ± 5%) and illumination (12 h light/dark cycle, lights on at 07h00). The noise level was maintained at less than 65 decibels. The animals were allowed access to food and fluids ad libitum. All animal experimentation was approved by the Animal Research Ethics Committee of the University of KwaZulu-Natal (Ethical clearance number: AREC/060/017D). Procedures involving animal care were conducted in conformity with the institutional guidelines for animal care of the University of KwaZulu-Natal.

### Experimental protocol

Using a previously described protocol, prediabetes was induced in experimental animals by continuously exposing them to a high-fat high-carbohydrate diet supplemented with 15% fructose for 20 weeks^15^. Meanwhile, the non-diabetic animals were given standard rat chow and water during the same 20-week period. At the end of week 20, the animals were randomly assigned to the following groups (n = 6 per group): non-diabetic (ND), prediabetic (PD), intermittently exercising PD (PD + IE) and regularly exercising PD (PD + RE). The animals remained on their respective diets until the end of experimentation. Treadmill exercise was for 7 weeks starting on week 21. The protocol duration for the ND and PD groups was the same as the other groups except that they had no access to a treadmill. Oral glucose tolerance (OGT) test, Novel Object Recognition Test (NORT) and the Morris-Water Maze (MWM) task assessments started one-week post-exercise cessation (week 29)^56^. The NORT was conducted on the first 3 days of week 29, the MWM task was done on the remaining 4 days of week 29 and the first 3 days of week 30 while the OGT test was performed on the 5^th^ day of week 30. Animals were sacrificed at the end of the experimental period, a day after the tests were completed after which prefrontal cortex (PFC) and hippocampal tissue were harvested for analysis of MDA, GPx1, IL-1β and dopamine concentration. Tissues collected was stored at -80°c in a bio freezer until ready for use.

### Exercise treadmill running protocol

The exercising animals were subjected to a rat treadmill apparatus consisting of a 2-lane animal exerciser for a 7-week exercise protocol^57^. The first 2 weeks were used as treadmill running acclimatization. During this period, the animals were familiarized with the treadmill apparatus by placing them on the moving treadmill every 3rd day before the actual treadmill running protocol. The training during the acclimatization period was gradually increased from 5 min to a maximum of 15 min duration at a constant running speed of 16 m/min. Thereafter, the animals were subjected to either an intermittent or regular treadmill running regimen for 5 weeks^58^. Both exercise regimens were of 15 min in duration and continuous but divided into three sessions of 5 min each with a 1 min rest period in between to prevent fatigue. The intermittent treadmill running regimen was performed every other 3rd day. For the PD + RE group, a program of regular treadmill running was performed every 24hrs at the same time for 5 weeks. The initial running speed for both intermittent and regular treadmill running regimen was set at 18 m/min and increased by 2 m/min every week for 5 weeks to a maximum of 26 m/min after 5 weeks. Necessary precautionary measures to prevent injuries were taken and constant surveillance of animals was done under the supervision of the Biomedical Research Unit personnel.

### OGT response

To determine the glucose tolerance response of animals following exercise during prolonged ingestion of a high-fat high-carbohydrate diet, an oral glucose tolerance test was conducted following carbohydrate loading. The OGT response of all animal groups was monitored in the animals using an established laboratory protocol^59^. Briefly, after an 18 h fasting period, glucose was measured (time 0) followed by loading with a monosaccharide syrup (glucose; 0.86 g/kg, p.o.) by oral gavage using a 38 mm long, 18-gauge gavage curved needle with a 21/4 mm ball end (Able Scientific, Canning Vale, Australia). To measure glucose concentration, blood was collected using the tail-prick method^60^. Glucose concentration was measured using an OneTouch select glucometer (LifeScan, Mosta, Malta, United Kingdom). Glucose concentration was measured at 15, 30, 60, and 120 min following carbohydrate loading.

### The novel object recognition test

The NORT was used to evaluate the animal’s ability to recognize a novel object in the environment one week after exercise treatment following voluntary ingestion of the high-fat high-carbohydrate diet. An open-field arena (90 cm length × 60 cm width × 35 cm height) was used as the NORT apparatus. The test procedure was carried out over 3 days and consisted of three phases: habituation, familiarization and test phase. In the habituation phase, each animal could freely explore the open-field arena in the absence of objects for 5 min. It was then removed from the arena and placed back in its holding cage. After 24 h, each animal was put back in the open-field arena containing two identical sample objects [A + A] for 10 min to familiarize with the objects. To prevent coercion to explore the objects, the animal was placed on the corner opposite to where the objects were, with its back to the objects. After a 24 h retention interval, the test phase was conducted where each animal was returned to the open-field arena with two objects, one familiar (object identical to previous objects) and one novel (object different to former objects) [A + B] for 10 min to assess the ability of the animal to recognize a novel object. During both the familiarization and test phase, to eliminate bias, the objects were centrally placed on the floor of the arena opposite to where the animals were. The objects were placed at an equal distance of 15 cm from each wall. The placing of the novel and the familiar object was counterbalanced and alternated throughout the test phase to eliminate the preference of sides in the room by the animals. After each session, the arena and objects were cleaned with 75% ethanol to ensure that the behaviour of animals was not influenced by odour cues. The test was video-recorded, and time spent exploring the objects was manually analyzed by the researcher and an evaluator blind to the study. Exploration of an object was defined as directing the nose at a distance ≤ 2 cm to an object, orientation of animal’s snout toward an object, sniffing or touching with the snout^61^. To determine discrimination between the novel and familiar objects, Discrimination Index (DI) was calculated in the following manner^61^:

$$DI=\frac{time\,spent\,exploring\,the\,novel\,object-time\,spent\,exploring\,the\,familiar\,object}{time\,spent\,exploring\,the\,novel\,object+time\,spent\,exploring\,the\,familiar\,object}$$

To determine novel object recognition, Recognition Index (RI) was calculated in the following manner^61^:

$$RI=\frac{time\,spent\,exploring\,the\,novel\,object}{time\,spent\,exploring\,the\,novel\,object+time\,spent\,exploring\,the\,familiar\,object}$$

### Morris-Water maze protocol

The MWM test was performed to assess spatial learning and memory^62^ in animals one week-post exercise cessation during voluntary ingestion of a high-fat high-carbohydrate diet. The MWM apparatus was a 100 cm diameter circular tank that was filled with water at an ambient temperature range between 22–23 °C up to the 24 cm mark. The tank was then divided into 4 equal quadrants that had at the centre of the wall’s visual cues of different colours and shapes, placed equidistant from each other. A hidden square plexiglass platform of 10 cm length × 10 cm width and 20 cm height submerged 4 cm under water and located in parallel to a visual cue of a quadrant but 27 cm from the perimeter was used as an escape/target platform. This was referred to as the goal quadrant^63^. The learning trials were conducted over 5 consecutive days, once in each quadrant per day with the interval between trials varying from 10–15 min^62,64^. After being dropped by tail-end in the water facing the tank wall, an animal had to learn to use distal visual cues to navigate a direct path to the hidden platform when started from different random locations of visual cues around the perimeter of the tank^62^. An animal had to find the platform within 2 min after which it could stay on for 1 min before being removed from the tank. If an animal failed to find the platform within the allotted 2 min, it was guided to the platform and allowed to stay on for 1 min. The time taken by an animal to find the hidden platform after being dropped in water is referred to as latency. The latency time was recorded throughout the procedure to assess the learning process. A probe test was conducted without the platform in the tank to assess the ability of an animal to recall the goal quadrant following a 24 h washout period after the last learning trial. An animal was placed in a novel start position, facing the tank wall and allowed to navigate around for 2 min before being removed from the tank. No animal was dropped within the goal quadrant and quadrants were alternated to minimize the bias of path preference in the animals. The probe test was video-recorded and manually analyzed by the researcher and an evaluator blind to the study.

### Tissue harvesting

At the end of the experimental period, all animals were decapitated using a sharp guillotine. Thereafter, the brain was removed immediately and placed in a frozen 0.9% saline slush to suppress the degradation of brain structures during dissection. The PFC and hippocampus were removed and collected into weighed and pre-cooled Eppendorf tubes. The tissues in the tubes were weighed and immediately snap-frozen in liquid nitrogen before storing at − 80 °C in a Bio Ultra freezer (Snijders Scientific, Holland) until the day of biochemical analysis.

### Biochemical analysis

PFC and hippocampal tissue GPx1, IL-1β, and dopamine concentration were measured using relevant ELISA kits (Elabscience Biotechnology Co., Ltd) according to the manufacturer’s instructions. PFC and hippocampal MDA concentration were measured using an established laboratory protocol^65^. Briefly, PFC or hippocampal tissue (50 mg) were homogenized in 0.2% phosphoric acid (500 μl). The homogenate was centrifuged at 400 × g for 10 min. Thereafter, the homogenate (400 μL) was added to 2% phosphoric acid (400 µL) and then separated into two glass tubes, each receiving equal volumes of the solution. Subsequently, 7% phosphoric acid (200 μL) was added into each glass tube followed by the addition of 400 μL of thiobarbituric acid (TBA)/butylated hydroxytoluene (BHT) into one glass tube (sample test) and 400 μL of 3 mM hydrochloric acid (HCl) into the second glass tube (blank).To ensure an acidic pH of 1.5, 1 M HCl (200 μL) was added into the sample and blank test tubes. Both solutions were heated at 100 °C for 15 min in a water bath and then allowed to cool to room temperature. Butanol (1.5 mL) was then added to each cooled solution. The solutions were vortexed for 1 min to ensure vigorous mixing and allowed to settle until 2 phases could be distinguished. The butanol phase (top layer) was transferred to Eppendorf tubes and centrifuged at 13,200×g for 6 min. The blanks and the samples were aliquoted into a 96-well microtiter plate in triplicate and the absorbance read at 532 nm (reference λ 600 nm) on a BioTek μQuant spectrophotometer (Biotek 42 Johannesburg, South Africa). The absorbance from these wavelengths was used to calculate the concentration of MDA using Beer’s Law^66^:

$$ [MDA]\,({\text{nmol}}\,{\text{g}}^{{ - 1}} ) = Average\,absorbance/Absorption\,coefficient\,(156\,{\text{mM}}^{{ - 1}} ). $$

### Analysis of data

All data were expressed as means ± S.E.M. Statistical comparisons were performed with GraphPad InStat software (version 5.00, GraphPad Software, Inc., San Diego, California, USA) using one-way analysis of variance (ANOVA) followed by the Tukey–Kramer post hoc multiple comparisons test or two-way ANOVA followed by Bonferroni comparison test. Correlation analysis between AUC_glucose_ and cognitive performance was performed using a Pearson test with individual data across the groups (groups = 4 × number of animals (6, per group) = 24). A value of *p* < 0.05 was considered statistically significant.

### Ethics approval

All animal experimentation was approved by the Animal Research Ethics Committee of the University of KwaZulu-Natal (Ethical Clearance Number: AREC/060/017D).

## Data Availability

The datasets used and/or analyzed during the current study are available from the corresponding author on reasonable request.

## Abbreviations

BHT: Butylated hydroxytoluene
DI: Discrimination index
DA: Dopamine
GPx1: Glutathione peroxidase
HFHC: High-fat high-carbohydrate
IL-1β: Interleukin-1β
PD + IE: Intermittently exercising PD
MDA: Malonaldehyde
MWM: Morris-Water maze
nAChR: Nicotinic acetylcholine receptors
ND: Non-diabetic
NORT: Novel object recognition test
ANOVA: One-way analysis of variance
OGT: Oral glucose tolerance
PD: Prediabetic
PFC: Prefrontal cortex
RI: Recognition index
PD + RE: Regularly exercising PD
TBA: Thiobarbituric acid
